# Ruptured middle cranial fossa arachnoid cysts after minor trauma in adolescent boys presenting with subdural hygroma: two case reports

**DOI:** 10.1186/s13256-021-03106-8

**Published:** 2021-10-12

**Authors:** Mohammad Farouq Hamidi, Hidayatullah Hamidi

**Affiliations:** 1 Neonatology Department, Maiwand Teaching Hospital, Kabul Medical University of Science (KMUS), Kabul, Afghanistan; 2 Radiology Department, French Medical Institute for Mothers and Children (FMIC), Kabul, Afghanistan

**Keywords:** Arachnoid cyst, Subdural hygroma, Ruptured arachnoid cyst, Case report

## Abstract

**Background:**

Intracranial arachnoid cysts are common, cerebrospinal fluid-filled, innocent lesions that are usually detected incidentally on brain imaging. They may rupture and complicate due to subdural hematoma or hygroma after minor trauma.

**Case summary:**

Authors present two cases of ruptured middle cranial fossa arachnoid cysts in adolescent (12-year-old and 15-year-old) Afghan boys presenting with subdural hygroma after minor trauma.

**Conclusion:**

Imaging work-up is necessary for symptomatic patients following minor head trauma as incidentally detected ruptured intracranial arachnoid cysts can be responsible for the symptoms.

## Introduction

Intracranial arachnoid cysts (IACs) are common, cerebrospinal fluid (CSF)-filled, innocent, congenital, benign, extra-axial intracranial lesions that are usually detected incidentally on brain imaging [1]. They may rupture and complicate due to subdural hematoma or hygroma after minor trauma. Imaging work-up is necessary for patients with signs and symptoms of increased intracranial pressure following head trauma.

## Case presentation

### Case 1

**Fig. 1:**
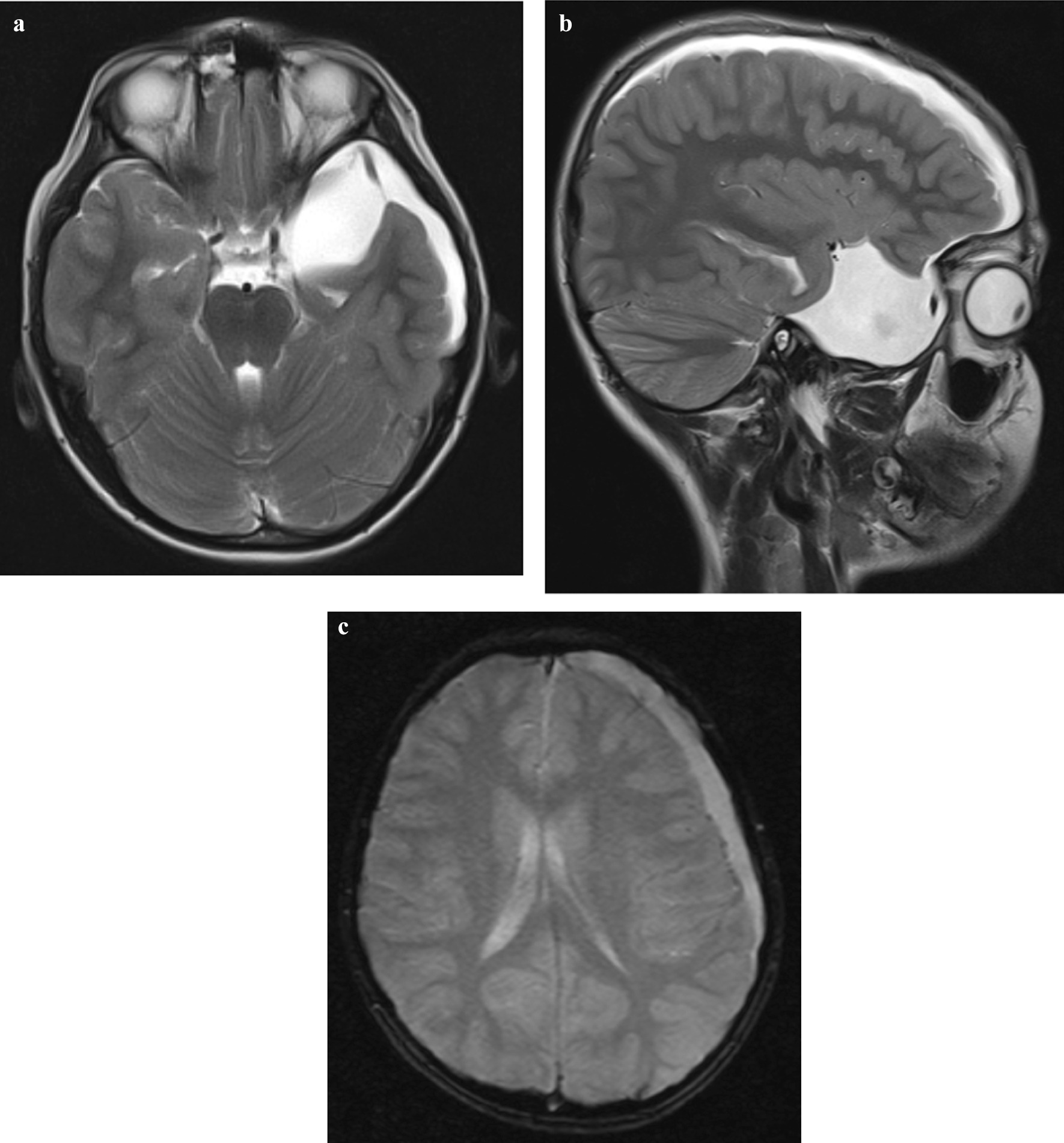
**a** Axial T2WI through the temporal lobes: CSF intensity lesion in the left middle cranial fossa anterior to left temporal lobe with mass effect. There is evidence of discontinuity in the peripheral wall of the cyst (black arrow) with subdural fluid in the vicinity of the temporal lobe (white arrow). **b** Sagittal T2WI through the left temporal lobe: CSF intensity lesion in the left middle cranial fossa anterior to left temporal lobe with subdural fluid extending along the convexity of the left cerebral hemisphere. **c** Axial T2* gradient recalled echo (GRE) sequence through the frontal lobes: the subdural fluid shows no blooming artifact to suggest hemorrhage, hence confirming the collection is CSF intensity (hygroma)

A previously healthy 12-year-old Afghan boy came to clinic complaining of a persistent headache for the last 20 days after minor head trauma while playing football. The patient also had nausea and vomiting for 4 days after trauma, which then stopped. No history of unconsciousness was documented. The Glasgow Coma Scale (GCS) of the patient was 15 at the time of presentation. No significant abnormal signs were detected on physical examination. Brain MRI was performed on day 21 post-trauma, which revealed a CSF intensity extra-axial lesion (arachnoid cyst) in left middle cranial fossa anterior to left temporal lobe (measuring 5 × 3.7 × 3.7 cm) with evidence of mass effect over left temporal lobe. CSF intensity fluid was seen in the subdural space along left cerebral hemisphere, representing subdural hygroma with evidence of continuity with the mentioned middle cranial fossa arachnoid cyst (Fig. 1a–c). No other gross abnormality was noted in the cranial fossa. Images were also remarkable for mucoperiosteal thickening of right maxillary, frontal, ethmoid, and sphenoid sinuses. The diagnosis of ruptured left middle cranial fossa arachnoid cyst with subdural hygroma was made. As the patient was clinically well and there was no significant sign of increased intracranial pressure at the time of imaging, the patient was treated conservatively and the headache gradually disappeared completely over a 3 month period of follow-up.

### Case 2

An ill-looking 15-year-old Afghan boy was brought to hospital complaining of vomiting, headache, and an episode of unconsciousness after head trauma in a road traffic accident 2 days ago. On physical examination, no abnormality was seen in the rest of the body system. No evidence of skull bruise or laceration was present.

**Fig. 2:**
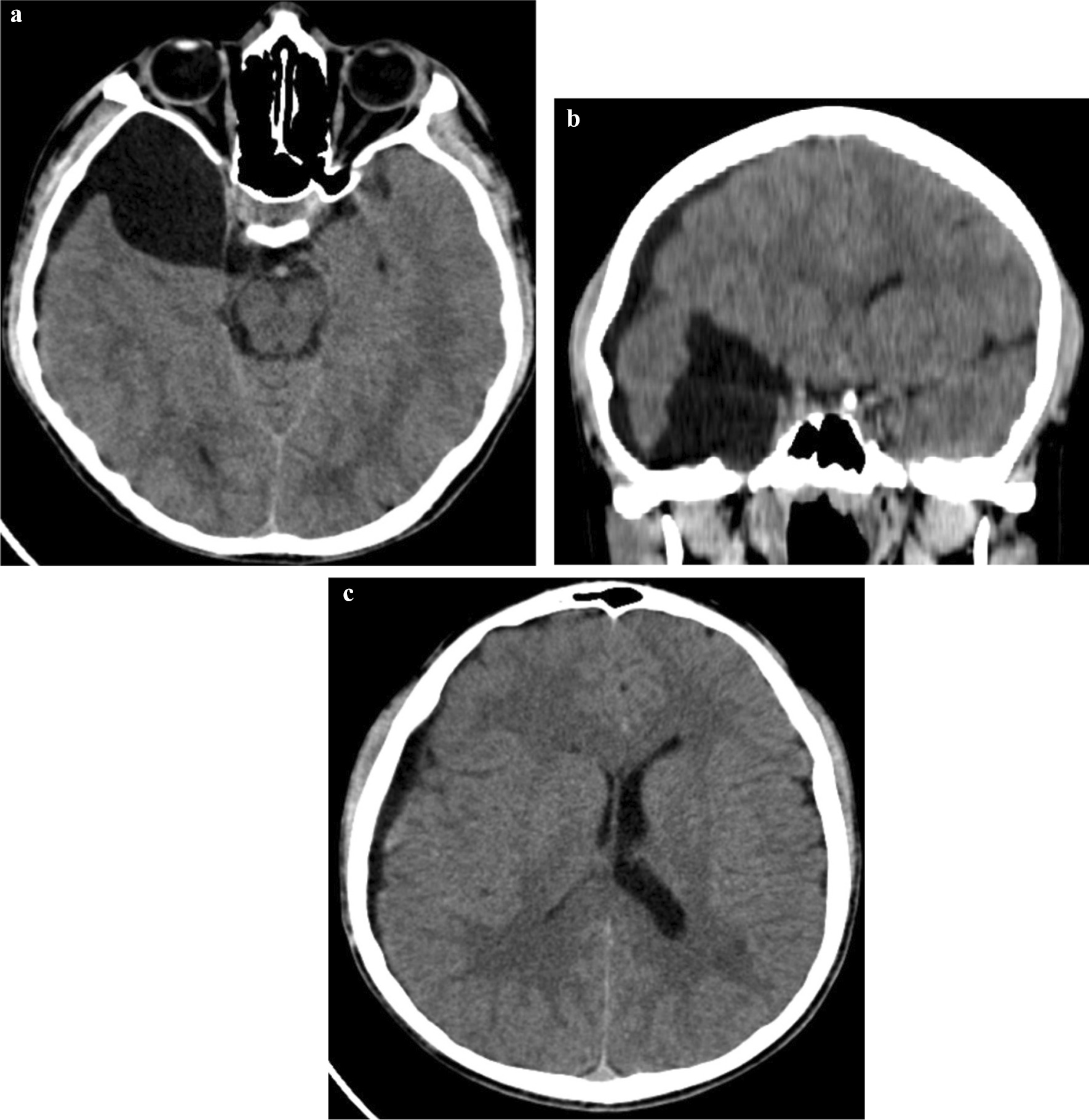
**a** Axial CT section through the temporal lobes: CSF intensity lesion in right middle cranial fossa anterior to left temporal lobe with mass effect. There is communication with subdural fluid in the vicinity of the temporal lobe. **b** Coronal CT section through the temporal lobes: CSF intensity lesion in right middle cranial fossa with atrophic changes of right temporal lobe. There is subdural fluid extending along the right frontotemporal lobes with resultant mass effect, midline shift, and effacement of right lateral ventricle. **c** Axial CT section at the level of lateral ventricles: subdural fluid along the right cerebral hemisphere, with mass effect, effacing the right lateral ventricle, mild midline shift, and prominent left lateral ventricle

Brain CT scan revealed a CSF intensity extra-axial lesion (arachnoid cyst) in the right middle cranial fossa anterior to right temporal lobe (measuring 5 × 5 × 4 cm) with evidence of atrophic changes of the temporal lobe and mass effect of the lesion. CSF intensity fluid (HU = 10) was seen in the subdural space (subdural hygroma) along right cerebral hemisphere with evidence of continuity with arachnoid cyst (Fig. 2a–c). No intracranial hematoma, hemorrhagic contusion, or skull bone fracture was present. The diagnosis of ruptured right middle cranial fossa arachnoid cyst with subdural hygroma was made. The patient was referred to the neurosurgery department and was lost to follow-up.

## Discussion and conclusions

IACs account for about 1% of all nontraumatic intracranial masses [1] with a predilection for male gender [2]. About 50–65% of IACs are located in the middle cranial fossa, more in the left side [2] (current cases were both in male patients; however, one was in the right and one in the left side). Other less common locations for intracranial arachnoid cysts are suprasellar cistern, quadrigeminal cisterns, cerebral convexities, cerebellopontine angles, and cisterna magna.

Middle cranial fossa IACs are often associated with variable degrees of temporal lobe agenesis and are classified into three types according to Galassi classification. Galassi type 1 cyst is small semicircular cyst confined to the anterior part of temporal fossa; type 2 is medium-sized, quadrangular cyst; and type 3 is large oval cyst with mass effect and midline shift [3]. The larger cysts are more likely to be symptomatic [4]. Symptoms can be generalized cranial enlargement, localized cranial bulging, increased intracranial pressure, seizures, psychomotor retardation, focal neurological deficits, hydrocephalus, and even dementia [5].

Occasionally, IACs may rupture—spontaneously or after trauma—and result in intracystic hemorrhage, subdural hematoma/subdural hygroma, or both [6], and may cause signs and symptoms of intracranial hypertension, mainly headache and vomiting [7]. Symptoms of IACs with subdural hygroma usually become increasingly severe for days or weeks after onset, but they eventually resolve [8].

A case–control study by Cress *et al.* on “risk factors for pediatric arachnoid cyst rupture/hemorrhage” demonstrated that children with larger intracranial arachnoid cysts (especially larger than 5 cm) were at increased risk of cyst rupture. A head injury within the previous 30 days, even if considered relatively minor, was also associated with cyst rupture [9]. These complications may occur at any age and are reported with a wide range of patients, from as young as 12 months to adults 53 years old [8, 10].

*Diagnosis* Computed tomography (CT) and magnetic resonance imaging (MRI) can provide the diagnosis of arachnoid cyst and its complications. On CT, IACs appear as well-defined lesions, with the same density of the CSF. MRI can further differentiate between chronic subdural hematoma and hygroma, which CT cannot do.

*Treatment and prognosis* Treatment of asymptomatic IACs is a matter of controversy. Symptomatic cysts are treated surgically, while disappearance of arachnoid cysts has also been reported, both with and without a history of preceding trauma [11]. Again, in cases with surgical treatment, the selection of the best neurosurgical approach also remains controversial. The options include cyst shunting, craniotomy for cyst fenestration, endoscopic fenestration, deviation of cyst fluid to another intracranial space, and even cystoperitoneal shunting [12, 13].

Regarding the management of complicated arachnoid cysts to subdural collections, the best surgical treatment remains controversial due to the lack of reported cases [2]. Slaviero *et al.* reported two cases to suggest that surgical evacuation of the hematoma followed by an endoscopic cyst fenestration was a minimally invasive, safe, and effective approach for the treatment of middle cranial fossa arachnoid cysts complicated with subdural collections [2]. Maher *et al.* reported a series of eight patients with IACs and subdural hygroma, of which seven were managed without surgery and only one underwent surgery. All patients experienced complete resolution of presenting signs and symptoms; however, half of them had objective findings strongly correlated with elevated ICP including cranial nerve (CN) VI, palsy, papilledema, and progressive macrocephaly [8]. Hence, they disagreed with the idea that surgical treatment of subdural hygroma associated with arachnoid cysts is always, or almost always, necessary; instead, they suggested that the decision to surgically treat symptomatic hygroma associated with arachnoid cysts should be made carefully. They claimed that symptoms of elevated ICP are not an absolute indication for surgical treatment [8].

## Conclusion

IACs are common, innocent, benign, usually incidentally detected intracranial lesions. They may rupture and complicate to subdural hematoma or hygroma after minor trauma. Imaging work-up is necessary for symptomatic patients following head trauma.

## Data Availability

Data sharing is not applicable to this article as no datasets were generated or analyzed during the current study (as this is a case report).

## Abbreviations

IACs: Intracranial arachnoid cysts
MRI: Magnetic resonance imaging
CT: Computed tomography
